# Characterization of tumour-infiltrating lymphocytes in a tumour rejection cynomolgus macaque model

**DOI:** 10.1038/s41598-020-65488-x

**Published:** 2020-05-21

**Authors:** Hiroki Satooka, Hirohito Ishigaki, Kagefumi Todo, Koji Terada, Yasutoshi Agata, Yasushi Itoh, Kazumasa Ogasawara, Takako Hirata

**Affiliations:** 1 Department of Fundamental Biosciences, Shiga University of Medical Science, Otsu, Japan; 2 Department of Pathology, Shiga University of Medical Science, Otsu, Japan; 3 Department of Biochemistry and Molecular Biology, Shiga University of Medical Science, Otsu, Japan

**Keywords:** Cancer models, Tumour immunology

## Abstract

Immunotherapy has emerged as a promising and effective treatment for cancer, yet the clinical benefit is still variable, in part due to insufficient accumulation of immune effector cells in the tumour microenvironment. Better understanding of tumour-infiltrating lymphocytes (TILs) from nonhuman primate tumours could provide insights into improving effector cell accumulation in tumour tissues during immunotherapy. Here, we characterize TILs in a cynomolgus macaque tumour model in which the tumours were infiltrated with CD4^+^ and CD8^+^ T cells and were eventually rejected. The majority of CD4^+^ and CD8^+^ TILs exhibited a CD45RA^−^CCR7^−^ effector memory phenotype, but unlike circulating T cells, they expressed CD69, a marker for tissue-resident memory T (T_RM_) cells. CD69-expressing CD8^+^ TILs expressed high levels of the cytotoxic molecule granzyme B and the co-inhibitory receptor PD-1. Consistent with the T_RM_ cell phenotype, CD8^+^ TILs minimally expressed CX3CR1 but expressed CXCR3 at higher levels than circulating CD8^+^ T cells. Meanwhile, CXCL9, CXCL10 and CXCL11, chemokine ligands for CXCR3, were expressed at high levels in the tumours, thus attracting CXCR3^+^CD8^+^ T cells. These results indicate that tumour-transplanted macaques can be a useful preclinical model for studying and optimizing T cell accumulation in tumours for the development of new immunotherapies.

## Introduction

Increasing evidence has demonstrated the important role of the immune system in controlling tumour development^1,2^. Strategies harnessing the immune system to treat cancer have made great advances in recent years^3^. These immunotherapies include cancer vaccines, immune checkpoint inhibitors and adoptive cell therapy. The successful use of immune checkpoint inhibitors, which target CTLA-4 and the PD-1/PD-L1 axis, has brought hope for cure in several types of cancer^4^. In addition, adoptive transfer of naturally occurring or genetically engineered T cells has shown promise in some malignancies^5^. The development of these immune-based antitumour therapies necessitates an animal model with an immune system similar to that of humans. Although mouse tumour models have provided important insights into antitumour immune responses, substantial differences between the mouse and human immune systems hamper the clinical translation of the results obtained in mouse tumour models^6^. Thus, tumour models utilizing nonhuman primates, which are phylogenetically closer to humans than any other laboratory animals, are required for the development and optimization of new immunotherapies^7^.

The clinical success of all immunotherapies relies on the efficient migration and localization of immune effector cells, whether endogenous or adoptively transferred, to tumour target tissue^8^. This issue is critical when immunotherapies are targeted to solid tumours. Tumour-infiltrating lymphocyte (TIL) populations can be isolated from various tumours, and CD8^+^ T cells (cytotoxic T cells; CTLs) are the major subset of TILs that mediate tumour regression. It is becoming clear that TILs share phenotypic characteristics with tissue-resident memory T (T_RM_) cells, a recently discovered lineage of T cells that remain in tissues without recirculating through the blood^9^. T_RM_-like TILs can be found in various human tumours and mouse models, and the presence of these TILs is associated with a favourable prognosis^10^.

A critical step in the generation of tumour-associated T_RM_ cells is the trafficking of T cells into the tumour. Immune effector cell trafficking to the tumour microenvironment is a highly dynamic process involving a series of distinct steps that include rolling, adhesion, extravasation and migration within the tissue^11^. These steps are primarily mediated by adhesion molecules and chemokines. Many cancers have a complex network of chemokines and their receptors, which is a key determinant of TIL accumulation at the tumour site^12^. CXCR3 and its ligands CXCL9 and CXCL10 have been implicated in CTL infiltration into several types of cancer and are positively correlated with a better prognosis^13–16^. In addition to CXCR3, CCR1, CCR4 and CCR5 have been implicated in T cell infiltration to the tumour site^17^. As effective immunotherapy requires that CTLs physically contact tumours cells, chemokine–chemokine receptor expression profiles that determine CTL migratory behaviour can be exploited to develop immunotherapies with enhanced efficacy. However, CTL migratory profiles in nonhuman primates, such as rhesus and cynomolgus macaques, have not been reported.

We previously established a novel tumour transplantation model in cynomolgus macaques, in which induced pluripotent stem cell (iPSC)–derived tumour cells carrying a homozygous major histocompatibility complex (MHC) haplotype were injected into MHC-matched macaques^18^. Although these cells formed tumours when injected into immunodeficient NOG mice, they were immunologically rejected in MHC-matched macaques within 4–5 weeks. Thus, this model may be useful for analysing TILs that generally play major roles in the immunological rejection of tumours and optimizing the efficacy of new immunotherapies.

In this study, we report the characterization of TILs in this iPSC-derived tumour transplantation macaque model. CD8^+^ TILs that accumulated at the tumour site had T_RM_-like phenotypes and expressed high levels of CXCR3, and the tumour expressed CXCR3 ligands, mirroring the characteristics of TILs in human cancers. Thus, this macaque model proves useful for analysing and optimizing T cell trafficking and localization to the tumour site in the development of new immunotherapies.

## Results

### CD4^+^ and CD8^+^ T cells are preferentially recruited from blood into the tumour in a macaque tumour model

**Figure 1:**
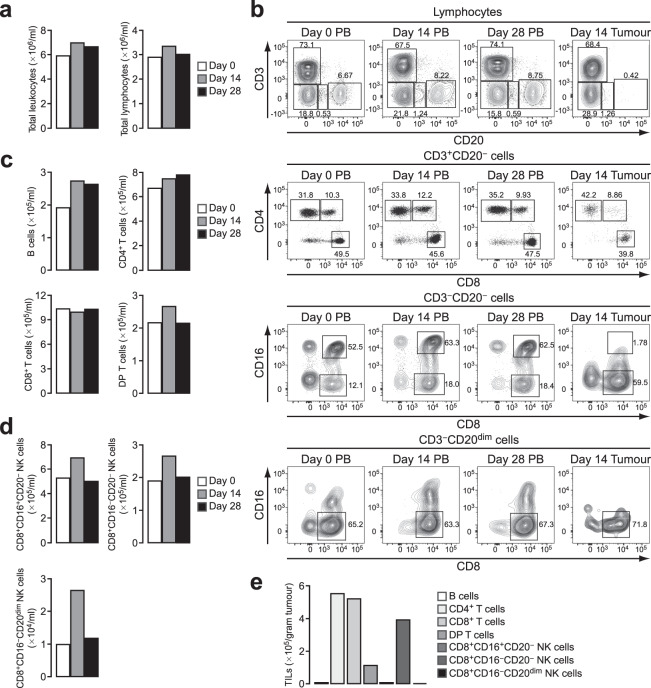
CD4^+^ and CD8^+^ T cells are the major TIL subsets in a cynomolgus macaque tumour model. (**a**) Total leukocyte and lymphocyte counts. Blood was obtained at days 0, 14 and 28 after tumour transplantation and analysed by flow cytometry. Live cells were gated as 7-AAD^−^. Leukocytes were determined as CD45^+^, and lymphocytes were gated according to forward and side scatter from the CD45^+^ cells. (**b**) Flow cytometry plots for the analysis of lymphocyte subsets in PB and tumour tissue. The tumour was resected at day 14 after transplantation. B cells (CD3^−^CD20^+^), CD4^+^ T cells (CD3^+^CD20^−^CD4^+^CD8^−^), CD8^+^ T cells (CD3^+^CD20^−^CD4^−^CD8^+^) and DP T cells (CD3^+^CD20^−^CD4^+^CD8^+^) were identified. Three subsets of NK cells were identified as CD3^−^CD20^−^CD8^+^CD16^+^, CD3^−^CD20^−^CD8^+^CD16^−^ and CD3^−^CD20^dim^CD8^+^CD16^−^. Numbers adjacent to the outlined areas indicate the percentage of cells in each. (**c**) Number of B cells, CD4^+^ T cells, CD8^+^ T cells and DP T cells in PB. (**d**) Number of the three NK cell subsets in PB. (**e**) Number of TIL populations in the tumour resected at day 14. The number of cells per gram tumour is shown.

We previously established tumour cell lines from iPSCs of a cynomolgus macaque carrying a homozygous MHC haplotype^18^. When these tumour cells, named PTY cells, were transplanted into cynomolgus macaques heterozygous for the matched MHC haplotype, they were rejected within 4–5 weeks. The involvement of humoral immunity as a mechanism of tumour rejection has been shown previously^18^, but the role of TILs in this model has not been investigated. TILs, together with various types of immune cells and stromal cells, contribute to the formation of a complex tumour microenvironment, which regulates tumour progression^19,20^. We first examined the peripheral blood (PB) lymphocyte compartment after tumour transplantation in comparison with TILs. PTY cells were injected into four separate regions of the backs of two macaques heterozygous for the matched MHC haplotype at day 0, and the generated tumours were surgically removed at day 14 before they were rejected. We monitored PB until day 28. Flow cytometric analysis revealed that during this time period, there were no apparent changes in total leukocyte and lymphocyte numbers of the first tumour-transplanted macaque (Fig. 1a). A slight increase in CD20^+^ B cells was observed at days 14 and 28 (Fig. 1b, the uppermost panel, and c). Within CD3^+^ T cell populations, CD4^+^CD8^−^ cells (CD4^+^ T cells), CD4^−^CD8^+^ cells (CD8^+^ T cells) and CD4^+^CD8^+^ double-positive T cells (DP T cells) were identified (Fig. 1b, the second upper panel). As reported previously^21^, DP T cells were found at a considerable percentage in cynomolgus macaque blood. The number of these T cell subsets did not change appreciably after tumour transplantation (Fig. 1c). As reported for the blood of rhesus macaques^22^, natural killer (NK) cells were identified as CD8^+^CD16^+^ and CD8^+^CD16^−^ cells within the CD3^−^CD20^−^ population and as CD8^+^CD16^−^ cells within the CD3^−^CD20^dim^ population (Fig. 1b, the bottom two panels). All of these NK cell subsets showed an increase in number at day 14 post injection (Fig. 1d).

To examine the composition of TIL populations in this model, single-cell suspensions were prepared from the tumour resected at day 14 post injection and were analysed by flow cytometry for the same markers used in the PB analysis (Fig. 1b). Most of the infiltrated lymphocytes were CD3^+^ T cells, while CD20^+^ B cells were found only marginally (Fig. 1e). Among T cell populations, CD4^+^ and CD8^+^ T cells were the major subsets. DP T cells were also found in the tumour, albeit less frequently than in PB. These results suggested that among the three circulating T cell populations, CD4^+^ and CD8^+^ T cells were preferentially recruited from blood into the tumour microenvironment. Similar results were obtained from the PB and tumour of the second tumour-transplanted macaque (Supplementary Fig. 1). Additionally, NK cells of the CD8^+^CD16^−^CD20^−^ phenotype were found in a considerable number in the tumour (Fig. 1e).

### The majority of CD4^+^ and CD8^+^ TILs express the T_RM_ marker CD69

**Figure 2:**
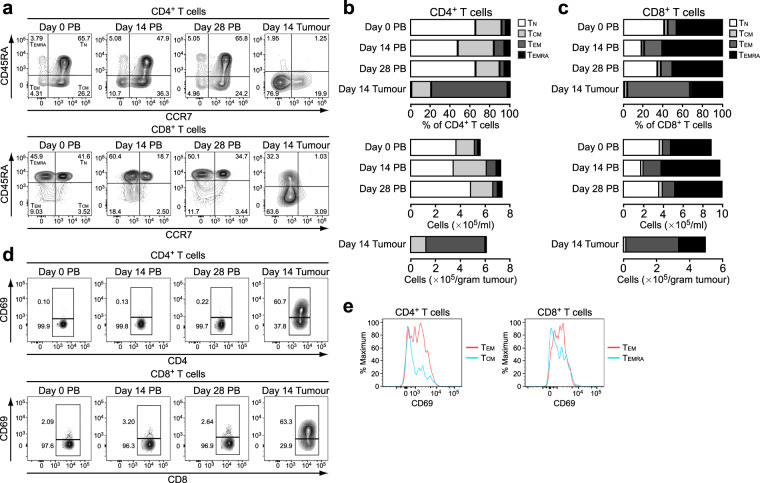
The majority of TILs are of the T_EM_ phenotype and express CD69. (**a**) Expression of CCR7 and CD45RA in CD4^+^ and CD8^+^ T cells from PB and tumour tissue. Blood was obtained at days 0, 14 and 28, and the tumour was resected at day 14 after tumour transplantation. The T_N_, T_CM_, T_EM_ and T_EMRA_ subsets were determined as CD45RA^+^CCR7^+^, CD45RA^−^CCR7^+^, CD45RA^−^CCR7^−^ and CD45RA^+^CCR7^−^, respectively, for both CD4^+^ and CD8^+^ T cells. Numbers in quadrants indicate the percentage of cells in each. (**b**) Percentage and number of T_N_, T_CM_, T_EM_ and T_EMRA_ subsets in CD4^+^ T cells from PB and tumour tissue. (**c**) Percentage and number of T_N_, T_CM_, T_EM_ and T_EMRA_ subsets in CD8^+^ T cells from PB and tumour tissue. (**d**) Expression of CD69 in CD4^+^ and CD8^+^ T cells from PB and tumour tissue. Numbers adjacent to the outlined areas indicate the percentage of cells in each. (**e**) Expression of CD69 in CD4^+^ T_EM_ and T_CM_ cells and in CD8^+^ T_EM_ and T_EMRA_ cells from tumours.

Because CD4^+^ and CD8^+^ T cells were the subsets most abundantly infiltrated in the tumour, we further examined these T cell compartments. Human CD4^+^ and CD8^+^ T cells in the blood and secondary lymphoid organs can be separated into four functionally different populations based on the expression of CD45RA and CCR7: naive (T_N_; CD45RA^+^CCR7^+^), central memory (T_CM_; CD45RA^−^CCR7^+^), effector memory (T_EM_; CD45RA^−^CCR7^−^) and effector memory re-expressing CD45RA (T_EMRA_; CD45RA^+^CCR7^−^)^23^. Similarly, the four populations were identified for both CD4^+^ and CD8^+^ T cell compartments in macaque blood (Fig. 2a). In the CD4^+^ T cell compartment in the blood, the percentage and number of T_CM_ and T_EM_ cells were increased at day 14, and these two subsets primarily constituted the CD4^+^ TILs (Fig. 2b). Among CD8^+^ T cell populations in the blood, T_EM_ and T_EMRA_ cells were increased at day 14 both in percentage and number, and these were the major subsets found in the tumour (Fig. 2c). For both CD4^+^ and CD8^+^ TILs, T_EM_ cells were the more abundant subset.

It has been shown that a subset of memory T cells does not recirculate but is maintained in peripheral tissues. Increasing evidence indicates that such T_RM_ cells accumulate in various human cancer tissues and constitute a substantial population of TILs^10^. Indeed, in this macaque model, CD69, a marker for T_RM_ cells, was barely expressed in circulating CD4^+^ and CD8^+^ T cells but was expressed in 56% and 64% of CD4^+^ and CD8^+^ TILs, respectively (Fig. 2d). Although CD69 is also a classical early marker of lymphocyte activation, CD69 expression on TILs was retained after culture with low-dose IL-2 (Supplementary Fig. 2), suggesting that CD69^+^ TILs are T_RM_ cells rather than early-activated lymphocytes. CD69-expressing cells were more frequently found in the T_EM_ subset than in the other subset for both CD4^+^ and CD8^+^ TILs (Fig. 2e). These results indicate that CD4^+^ and CD8^+^ T_RM_-like cells accumulated in the tumour tissue in this model.

### T_RM_-like CD8^+^ TILs express high levels of granzyme B

**Figure 3:**
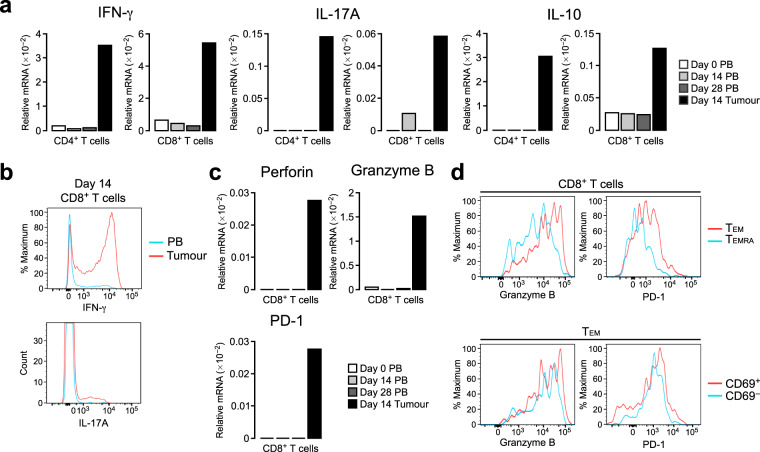
CD8^+^ TILs express high levels of cytotoxic molecules and PD-1. (**a**) Relative mRNA expression of IFN-γ, IL-17A and IL-10 in CD4^+^ and CD8^+^ T cells isolated from PB and tumour tissue. Blood was obtained at days 0, 14 and 28, and the tumour was resected at day 14 after tumour transplantation. The mRNA levels were assessed by real-time PCR and are presented relative to β-actin levels. (**b**) Intracellular staining for IFN-γ and IL-17A in CD8^+^ T cells from PB and tumours. Blood and the tumour were obtained at day 14. (**c**) Relative mRNA expression of perforin, granzyme B and PD-1 in CD8^+^ T cells isolated from PB and tumour tissue. The mRNA levels were assessed as in (**a**). (**d**) Flow cytometric analysis of the expression of granzyme B and PD-1 in CD8^+^ T cells and CD8^+^ T_EM_ cells from tumours.

To characterize the functional properties of TILs in this macaque tumour model, we sorted CD4^+^ and CD8^+^ T cells from the tumour tissue as well as from PB and quantified the mRNA levels of cytokines in these populations. In both CD4^+^ and CD8^+^ TILs, IFN-γ was markedly increased compared to that in blood CD4^+^ and CD8^+^ T cells, respectively, suggesting that TILs contain IFN-γ–producing T_H_1 cells and CTLs (Fig. 3a). IL-17A, a cytokine produced primarily by T_H_17 cells, was also remarkably increased in CD4^+^ TILs. CD8^+^ TILs also expressed IL-17A, albeit less than CD4^+^ TILs. Intracellular cytokine staining also showed that CD8^+^ TILs contained more IFN-γ–producing cells and IL-17A–producing cells than blood CD8^+^ T cells (Fig. 3b). In addition to inflammatory cytokines, the anti-inflammatory cytokine IL-10 was expressed at a high level specifically in CD4^+^ TILs (Fig. 3a), suggesting that CD4^+^ TILs contain a population of regulatory T cells.

Infiltrated CTLs express several cytotoxic molecules such as perforin and granzyme B, which lyse tumour cells. In this macaque model, CD8^+^ TILs showed much higher expression levels of perforin and granzyme B compared to CD8^+^ T cells in the blood (Fig. 3c). Flow cytometric analysis of CD8^+^ TILs revealed that T_EM_ cells, particularly CD69-expressing T_RM_-like cells, expressed high levels of granzyme B (Fig. 3d). CD8^+^ TILs often express high levels of the co-inhibitory receptor PD-1, and the expression status of PD-1 on TILs predicts the response to anti–PD-1 therapy^24,25^. Quantitative PCR analysis revealed that PD-1 mRNA expression was significantly increased in CD8^+^ TILs compared to blood CD8^+^ T cells (Fig. 3c). Among CD8^+^ TILs, T_EM_ cells expressed higher levels of PD-1 than T_EMRA_ cells, and the CD69-expressing subset contained cells with the highest levels of PD-1 (Fig. 3d). Taken together, these results suggested that the CD8^+^ TILs with the T_RM_-like phenotype showed the highest cytotoxic properties, although they also expressed high amounts of PD-1. Additionally, the DP T cell subset in the tumour, which comprised primarily of T_EM_ and CD69-expressing cells, contained cells with high levels of granzyme B (Supplementary Fig. 3), suggesting that T_RM_-like DP T cells also have cytotoxic properties.

### CXCR3 and its ligands are upregulated in the tumour microenvironment

**Figure 4:**
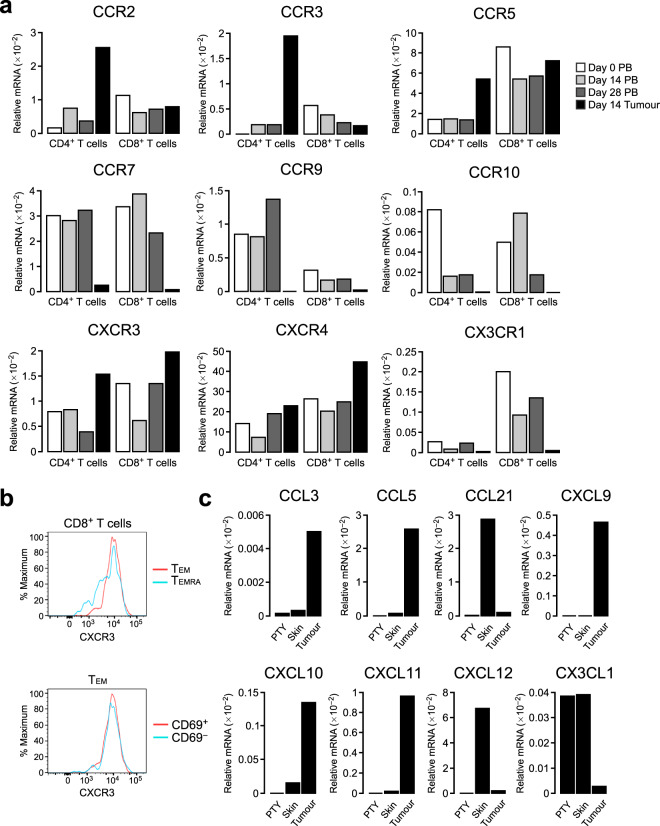
CXCR3 and its ligands are upregulated in the tumour microenvironment. (**a**) Relative mRNA expression of chemokine receptors in CD4^+^ and CD8^+^ T cells isolated from PB and tumour tissue. The mRNA levels were assessed by real-time PCR and are presented relative to β-actin levels. (**b**) Flow cytometric analysis of CXCR3 expression in CD8^+^ T cells and CD8^+^ T_EM_ cells from tumours. (**c**) Relative expression of chemokines in cultured PTY cells, skin and tumour. The mRNA levels were assessed as in (**a**).

The chemokine–chemokine receptor system is one of the major factors governing the trafficking of immune cells from blood to tissues, including tumour tissue^12^. To explore the chemokines and chemokine receptors involved in T cell trafficking from blood to the tumour in this macaque model, we quantified the mRNA levels of all known chemokine receptors in CD4^+^ and CD8^+^ T cells sorted from the tumour tissue and PB. CCR2, CCR3, CCR5, CXCR3 and CXCR4 were upregulated in CD4^+^ TILs compared to blood CD4^+^ T cells, while CCR7, CCR9, CCR10 and CX3CR1 were downregulated (Fig. 4a). CXCR3 and CXCR4 were upregulated in CD8^+^ TILs compared to blood CD8^+^ T cells at day 14, with CXCR3 showing more prominent upregulation than that in CD4^+^ T cells. Similar to CD4^+^ TILs, CCR7, CCR9, CCR10 and CX3CR1 were markedly downregulated in CD8^+^ TILs compared to blood CD8^+^ T cells. The low CCR7 mRNA levels were in agreement with the small percentage of CCR7^+^ T_N_ and T_CM_ cells in both CD4^+^ and CD8^+^ TILs (Fig. 2a–c). Flow cytometric analysis showed that CXCR3 was expressed on CD8^+^ TILs at slightly higher levels on T_EM_ than T_EMRA_ cells, while similar CXCR3 expression levels were observed between CD69^+^ and CD69^−^ cells (Fig. 4b).

We next examined the mRNA expression of chemokines in the tumour tissue and compared the mRNA levels with those in cultured PTY cells and in the skin before tumour injection. Among the chemokine ligands tested, the CCR5 ligands CCL3 and CCL5 were remarkably upregulated in the tumour tissue compared to PTY cells or control skin (Fig. 4c). CCL5 is also a ligand for CCR3. Thus, the CCL3/CCL5–CCR5 and CCL5–CCR3 axes appear to play a role in CD4^+^ T cell infiltration in the tumour. In contrast, the CCR7 ligand CCL21 was expressed at a lower level than the control skin, which was in agreement with the low expression of CCR7 in both CD4^+^ and CD8^+^ TILs. The CXCR3 ligands CXCL9, CXCL10 and CXCL11 were all elevated in the tumour, suggesting that the CXCL9/CXCL10/CXCL11–CXCR3 axis is involved in both CD4^+^ and CD8^+^ T cell infiltration into the tumour. The CXCR4 ligand CXCL12 and the CX3CR1 ligand CX3CL1 were downregulated in the tumour tissue.

### Repeated tumour injection induces rapid loss of CXCR3^high^ CD8^+^ T cells in the blood

**Figure 5:**
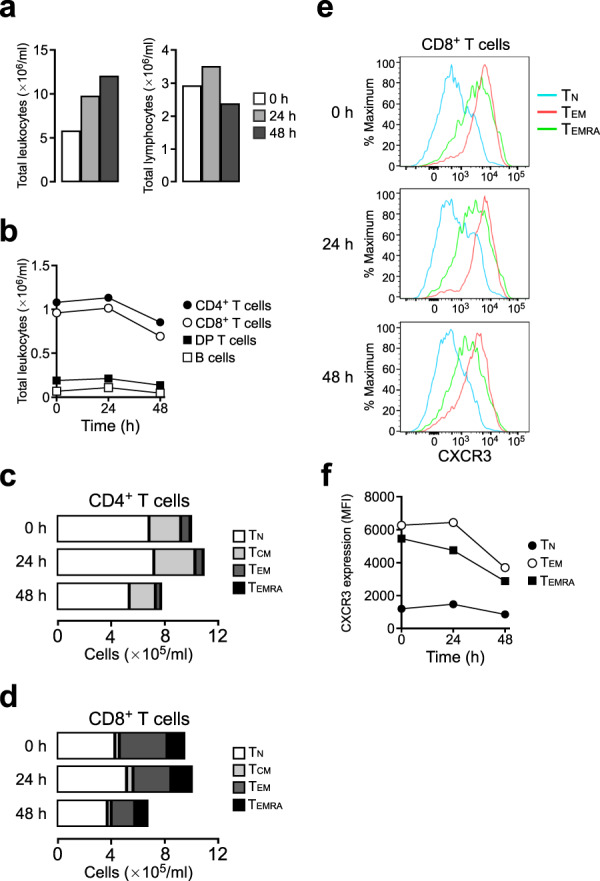
Repeated tumour injection induces rapid loss of CXCR3^high^ CD8^+^ T cells from blood. (**a**) Total leukocyte and lymphocyte counts in PB of a macaque repeatedly injected with tumour cells. Blood was obtained at 0, 24 and 48 h after tumour injection. (**b**) Number of lymphocyte subsets in PB. (**c**) Number of T_N_, T_CM_, T_EM_ and T_EMRA_ cells in CD4^+^ T cells from PB. (**d**) Number of T_N_, T_CM_, T_EM_ and T_EMRA_ cells in CD8^+^ T cells from PB. (**e**) Expression of CXCR3 on CD8^+^ T_N_, T_EM_ and T_EMRA_ cells in PB. (**f**) Time course of mean fluorescence intensity (MFI) of CXCR3 expression on CD8^+^ T_N_, T_EM_ and T_EMRA_ cells in PB.

When PTY cells were injected into a cynomolgus macaque for the first time, they were rejected within 4–5 weeks. However, repeated tumour cell injection into the same individual induced rapid tumour rejection without tumour growth, suggesting that tumour-specific lymphocytes had been expanded and were rapidly recruited from blood to the tumour injection site. To explore the involvement of CXCR3 in this repeated injection model, we examined PB by flow cytometry. The lymphocyte number in the blood was decreased at 48 h post injection (Fig. 5a), and both CD4^+^ and CD8^+^ T cell numbers were decreased (Fig. 5b). CD4^+^ and CD8^+^ T cell compartment analysis revealed that CD4^+^ T_CM_ and T_EM_ cells, the major subsets of CD4^+^ TILs, as well as CD8^+^ T_EM_ and T_EMRA_ cells, the major subsets of CD8^+^ TILs, were decreased at 48 h post injection (Fig. 5c,d). Compared to that in naive macaques, the CD8^+^ T_EM_ cell subset was remarkably expanded in the blood of the repeated injection model, and this subset was the one most substantially decreased at 48 h post injection. At this time point, CXCR3 expression in CD8^+^ T_EM_ and T_EMRA_ cells was appreciably decreased (Fig. 5e,f), suggesting that CXCR3^high^ cells had migrated into the tumour injection site. Together, these results support the view that CXCR3 plays a role in T cell migration into the tumour tissue in this macaque tumour model.

## Discussion

In this study, we showed that a nonhuman primate tumour model, in which iPSC-derived tumour cells were transplanted into MHC-matched macaques, represents a useful animal model for studying TILs. Chemokine–chemokine receptor expression profiles in TILs and tumour locales in this macaque model share many features with those reported for human cancers, rendering this model useful for preclinical studies that aim to optimize immune effector cell trafficking to the tumour site.

The chemokine–chemokine receptor system is a key regulator of TIL accumulation in the tumour environment^12^. We found that CD8^+^ TILs exhibited higher expression levels of CXCR3 compared to circulating CD8^+^ T cells. CXCL9, CXCL10 and CXCL11, chemokine ligands for CXCR3, were expressed at high levels in the tumour locale. These results are consistent with a recent report that CXCR3 is the major chemokine receptor required for CD8^+^ T cell migration into mouse and human melanoma^15^. Previously, CXCR3 expression in activated CD8^+^ T cells was reported to be associated with enhanced survival in melanoma patients with stage III disease^13^. In a mouse model of melanoma, CXCR3 deficiency attenuates the infiltration of CD8^+^ T cells into tumours and thereby reduces antitumour immunity^26^. CXCL9 and CXCL10 expression is associated with survival in human colorectal cancer^14^ and exerts tumour suppressive function by TIL recruitment in ovarian cancer^16^.

Accumulating evidence indicates that a substantial population of CD8^+^ TILs are T_RM_-like cells^10^. These T_RM_ cells have been identified by surface expression of CD103 and CD69 in both humans and mice. Indeed, the majority of CD8^+^ TILs in this macaque model expressed CD69. CD69 downregulates the sphingosine 1-phosphate receptor S1PR1, which is required for T cell egress from tissues and thus promotes tissue residency^27,28^. On the other hand, CD103 binds to the epithelial cell marker E-cadherin, thereby promoting T cell residency in epithelial tumours and antitumour CTL function^29,30^. In our model, CD103 expression was not detected in CD8^+^ TILs, which is likely due to the non-epithelial nature of PTY tumour cells. Human T_RM_ cells infiltrating the lungs express CXCR3, CXCR6 and CCR6 at higher levels compared to blood T_EM_ cells^31^, while in our macaque tumour model, CXCR3 but not CXCR6 or CCR6 was upregulated in CD8^+^ TILs. Consistent with the low expression of CX3CR1 mRNA in human T_RM_ cells, CX3CR1 expression was remarkably downregulated in CD8^+^ TILs in the macaque tumour model.

In recent years, immune checkpoint blockade has been revolutionizing cancer therapy. PD-1 inhibitors, such as nivolumab and pembrolizumab, have been approved for use in the treatment of melanoma and non-small cell lung cancer^32^. In the tumour microenvironment, the PD-1 and PD-L1 interaction supports tumour development by negatively regulating immune responses. PD-1 inhibitors block this interaction, leading to more robust T cell activity and improved prognosis. The decreased efficacy of PD-1 inhibitor treatment in CXCR3-deficient mice suggests that CXCR3-expressing CD8^+^ TILs are the responder cells in anti–PD-1 therapy^26^. In our macaque model, PD-1 expression was higher in CD8^+^ TILs compared to blood CD8^+^ T cells, and within the CD8^+^ TILs, CD69-expressing T_RM_-like cells expressed higher levels of PD-1 than CD69^−^ cells.

Although we focused mostly on CD4^+^ and CD8^+^ T cells in this paper, DP T cells and NK cells also infiltrated the tumour in this model. DP T cells in cynomolgus macaque blood are known to be CD8α^+^β^−^ extrathymic T cells^21^. In our analysis, blood DP T cells were mostly T_EMRA_ cells, but similar to CD4^+^ and CD8^+^ TILs, DP T cells in the tumour comprised primarily of T_EM_ cells. Interestingly, DP T cells in the tumour contained cells with high cytotoxic properties, suggesting that these cells also contributed to tumour rejection in this model. In this regard, DP T cells in cynomolgus macaque blood have cytotoxic activity comparable to that of CD8^+^ T cells^33^. NK cells infiltrating the tumour in this model were mostly of the CD8^+^CD16^−^CD20^−^ phenotype, while CD8^+^CD16^+^CD20^−^ NK cells were dominant in blood. Similarly, in human melanoma patients, CD56^dim^CD16^−^ cells are the dominant subset in tumour-infiltrating NK cells, while CD56^dim^CD16^+^ cells are dominant among circulating NK cells^34^. It is conceivable that NK cells also play a role in tumour rejection in this model.

Adoptive immunotherapy using naturally occurring or gene-engineered tumour-specific T cells also offers a promising cancer therapy. In particular, chimeric antigen receptor (CAR) T cells have been proven to serve as a powerful new class of adoptive immunotherapeutics. Adoptive immunotherapy for solid tumours requires that T cells traffic to the tumour tissue. Although T cell trafficking has been extensively studied in mouse tumour models, extrapolation of mouse data to humans is limited due to differences between the mouse and human immune systems^6^. Humanized mouse models can produce some of the human immune responses, yet they still cannot replicate the entire human immune response to tumours. Moreover, tumours develop in complex microenvironments that regulate tumour progression and metastasis and in turn the efficacy of immunotherapy. Nonhuman primates have immunologically closer microenvironments to human cancers than any other animal tumour model and thus can provide relevant models for testing the efficacy of antitumour therapies^7^. The understanding of chemokine receptor expression patterns of CD8^+^ TILs in macaque tumour models could be harnessed to enhance the targeting of T cells towards tumours.

Overall, this study shows that our macaque model represents a useful model for studying tumour rejection, which will be exploited to develop new anticancer therapies. The chemokine receptor expression patterns of CD8^+^ TILs in this model provide insights into the molecular targets that promote T cell infiltration and accumulation at the tumour site, which can be used to enhance the efficacy of adoptive immunotherapy for cancer.

## Methods

### Experimental animals and tumour transplantation

Cynomolgus macaques (*Macaca fascicularis*), both MHC homozygous and heterozygous for a particular set of Mafa haplotype alleles called HT1, were identified in the Filipino macaque population. The establishment of iPSCs from an MHC homozygous cynomolgus macaque, generation of iPSC-derived tumours cells (PTY cells) and transplantation of PTY cells to MHC heterozygous cynomolgus macaques were performed as described in the previous study^18^. In one experiment, PTY cells were repeatedly transplanted into the same individual macaque. All protocols for animal experiments were approved by the Shiga University of Medical Science Animal Experiment Committee. This study was carried out in strict accordance with the Guidelines for the Husbandry and Management of Laboratory Animals of the Research Center for Animal Life Science at Shiga University of Medical Science and the Fundamental Guidelines for Proper Conduct of Animal Experiments and Related Activities in Academic Research Institutions under the jurisdiction of the Ministry of Education, Culture, Sports, Science and Technology, Japan.

### Cell preparation

Leukocytes were isolated from PB after the lysis of red blood cells with ammonium-chloride-potassium (ACK) buffer. For TIL isolation, removed tumours were weighed, minced and then incubated in RPMI 1640 containing 2 mg/ml collagenase D (Roche) and 100 μg/ml DNase I (Roche) at 37 °C for 1 h. Digested samples were passed through a 70-μm nylon mesh twice and used as a mixture of TILs and tumour cells. The total cells were enumerated with a haemocytometer.

### Flow cytometry

The monoclonal antibodies (mAbs) used for flow cytometric analyses were purchased from BD Biosciences, eBioscience or BioLegend, and included antibodies to CD3 (SP34-2), CD4 (OKT4), CD8 (SK1 or RPA-T8), CD16 (3G8), CD20 (2H7), CD45 (D058-1283), CD45RA (5H9), CD69 (FN50), CCR7 (G043H7), CXCR3 (G025H7), granzyme B (GB11), IFN-γ (B27), IL-17A (BL168) and PD-1 (EH12.2H7). 7-Aminoactinomycin D (7-AAD) was used to distinguish between live and dead cells. Single-cell suspensions were incubated with human Fc receptor blocking reagent (Miltenyi Biotec) for 10 min, followed by staining with mAbs for 60 min on ice and washing. For intracellular cytokine staining, the cells were fixed and permeabilized using a BD Cytofix/Cytoperm Fixation/Permeabilization Kit. Data were acquired on a FACSAria or FACSCanto II (both from BD Biosciences) and analysed using FlowJo (Tree Star). From the total cell count and the percentage of each subset, the cell number of each subset was calculated. For sorting of CD4^+^ and CD8^+^ T cell subsets, lymphocytes were gated according to forward and side scatter from live (7-AAD^−^) CD45^+^ cells, and CD4^+^ T (CD3^+^CD4^+^CD8^−^CD20^−^) and CD8^+^ T (CD3^+^CD4^−^CD8^+^CD20^−^) cells were sorted from the lymphocyte gate using a FACSAria.

### Quantitative PCR

Total RNA was extracted using TRIzol reagent (Invitrogen) and reverse transcribed with a High Capacity RNA-to-cDNA Kit (Applied Biosystems). Quantitative real-time PCR was performed using KOD SYBR qPCR Mix (Toyobo) and a LightCycler 480 instrument (Roche). The primer pairs used are listed in Supplementary Table 1.

## Supplementary information

Supplementary Information.
