# Supplementary material for: Characterization of tumour-infiltrating lymphocytes in a tumour rejection cynomolgus macaque model
Source: Sci Rep. 2020 May 21;10:8414. doi: 10.1038/s41598-020-65488-x (PMC7242367; doi:10.1038/s41598-020-65488-x)
Supplement: Supplementary file 1 — Supplementary Information. [file 41598_2020_65488_MOESM1_ESM.pdf]

Supplementary Materials for:

Characterization of tumour-infiltrating lymphocytes in a tumour rejection cynomolgus macaque model

Hiroki Satooka[1], Hirohito Ishigaki[2], Kagefumi Todo[1], Koji Terada[3], Yasutoshi Agata[3], Yasushi Itoh[2], Kazumasa Ogasawara[2] & Takako Hirata[1]

[1]Department of Fundamental Biosciences, Shiga University of Medical Science, Otsu, Japan.

[2]Department of Pathology, Shiga University of Medical Science, Otsu, Japan.

[3]Department of Biochemistry and Molecular Biology, Shiga University of Medical Science, Otsu, Japan.

Supplementary Table 1. Sequences of primers used in quantitative PCR

Gene	Forward primer	Reverse primer
CCR1	5′-GGCTATGGAAGTGACGGAGG-3′	5′-CCTGTGGAACAACTGCCGTA-3′
CCR2	5′-CCACATCTCGTTCTCGGTTTATC-3′	5′-CAGGGAGCACCGTAATCATAATC-3′
CCR3	5′-GTCATCATGGCGGTGTTTTTC-3′	5′-CAGTGGGAGTAGGCGATCAC-3′
CCR4	5′-CCCACGGATATAGCAGACACC-3′	5′-GTGCAAGGCTTGGGGATACT-3′
CCR5	5′-TTCTGGGCTCCCTACAACATT-3′	5′-TTGGTCCAACCTGTTAGAGCTA-3′
CCR6	5′-TTCAGCGATGTTTTCGACTCC-3′	5′-GCAATCGGTACAAATAGCCTGG-3′
CCR7	5′-TGAGGTCACGGACGATTACAT-3′	5′-GTAGGCCCACGAAACAAATGAT-3′
CCR8	5′-CTGTCTGACCTGCTTTTTGTCT-3′	5′-CCACTTTGCACATTACAGTCCC-3′
CCR9	5′-ATGTCAGGCAGTTTGCGAG-3	5′-TGCAGTACCAGTAGACAAGGAT-3′
CCR10	5′-TGAAGAGGACGCATACTCGG-3′	5′-CCACGGTCAGGGAGACACT-3′
CXCR1	5′-CTGACCCAGAAGCGTCACTTG-3′	5′-CCAGGACCTCATAGCAAACTG-3′
CXCR2	5′-CCTGTCTTACTTTTCCGAAGGAC-3′	5′-TTGCTGTATTGTTGCCCATGT-3′
CXCR3	5′-TTTGACCGCTACCTGAACATAGT-3′	5′-GGGAAGTTGTATTGGCAGTGG-3′
CXCR4	5′-ACGCCACCAACAGTCAGAG-3′	5′-AGTCGGGAATAGTCAGCAGGA-3′
CXCR5	5′-CACGTTGCACCTTCTCCCAA-3′	5′-GGAATCCCGCCACATGGTAG-3′
CX3CR1	5′-AGTGTCACCGACATTTACCTCC-3′	5′-AAGGCGGTAGTGAATTTGCAC-3′
CCL3	5′-AGTTCTCTGCATCACTTGCTG-3′	5′-CGGCTTCGCTTGGTTAGGAA-3′
CCL4	5′-CTCCTTGTTCTAGGACAGACTAGC-3′	5′-GGGCAGGGGTGTGTCATCTC-3′
CCL5	5′-CCAGCAGTCGTCTTTGTCAC-3′	5′-CTCTGGGTTGGCACACACTT-3′
CCL7	5′-CCCTCCAACGTGAAAACCTCT-3′	5′-GTGGCTGCTGGTGATCCTTC-3′
CCL11	5′-CCCCTTCAGCGACTAGAGAG-3′	5′-TCTTGGGGTCGGCACAGAT-3′
CCL13	5′-CTCAACGTCCCATCTACTTGC-3′	5′-TCTTCAGGGTGTGAGCTTTCC-3′
CCL21	5′-GTTGCCTCAAGTACAGCCAAA	5′-AGAACAGGATAGCTGGGATGG-3′
CCL24	5′-GGAGTGGGTCCAGAGGTACAT-3′	5′-CAGGTGGTTTGGTTGCCAG-3′
CCL26	5′-GAAACCTGTGAAGGGCCTGA-3′	5′-GAAGGGACTTGTGGCTGTGT-3′
CXCL9	5′-TCAGCCAGTGACCAACCTTT-3′	5′-TCCACTAACCGACTTGGCTG-3′
CXCL10	5′-GTGGCATTCAAGGAGTACCTC-3′	5′-TGATGGCCTTCGATTCTGGATT-3′
CXCL11	5′-GACGCTGTCTTTGCATAGGC-3′	5′-GGATTTAGGCATCGTTGTCCTTT-3′
CXCL12	5′-ATTCTCAACACTCCAAACTGTGC-3′	5′-ACTTTAGCTTCGGGTCAATGC-3′
CX3CL1	5′-GCCACAGGCGAAAGCAGTA-3′	5′-GGAGGCACTCGGAAAAGCTC-3′
IFN-γ	5′-TCGGTAACTGACTTGAATGTCCA-3′	5′-TCGCTTCCCTGTTTTAGCTGC-3′
IL-17	5′-AGATTACTACAACCGATCCACCT-3′	5′-GGGGACAGAGTTCATGTGGTA-3′
IL-10	5′-GACTTTAAGGGTTACCTGGGTTG-3′	5′-TCACATGCGCCTTGATGTCTG-3′
Perforin	5′-GTGGGACAATAACAACCCCAT-3′	5′-TGGCATGATAGCGGAATTTTAGG-3′
Granzyme B	5′-TGTGAAAAGAGCCATCCCCC-3′	5′-GTATTTTCCATCGGGGGTCGT-3′
PD-1	5′-CCAGGATGGTTCTTAGACTCCC-3′	5′-TTTAGCACGAAGCTCTCCGAT-3′
β-actin	5′-ATATCGCCGCGCTCGTTGT-3′	5′-GATGCCGTGCTCGATAGGG-3′

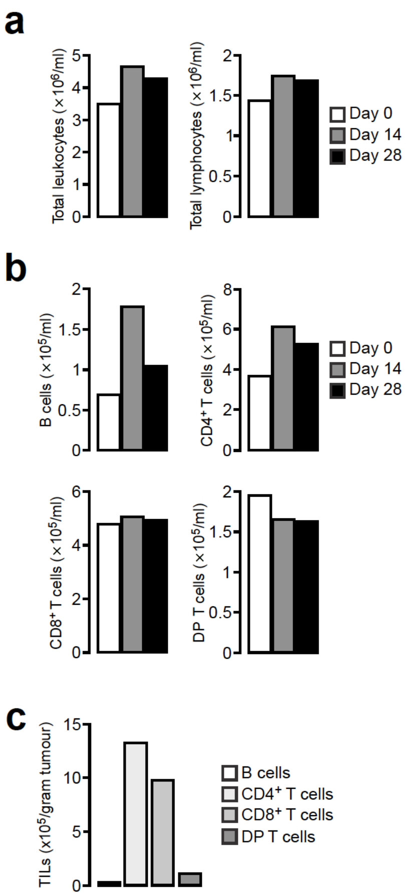

Supplementary Figure 1. $CD4^+$ and $CD8^+$ T cells are the major TIL subsets in the second tumour-transplanted cynomolgus macaque. (**a**) Total leukocyte and lymphocyte counts. Blood was obtained at days 0, 14 and 28 after tumour transplantation and analysed by flow cytometry. Live cells were gated as 7-AAD^-. Leukocytes were determined as $CD45^+$, and lymphocytes were gated according to forward and side scatter from the $CD45^+$ cells. (**b**) Number of B cells, $CD4^+$ T cells, $CD8^+$ T cells and DP T cells in PB. (**c**) Number of TIL populations in the tumour resected at day 14. The number of cells per gram tumour is shown.

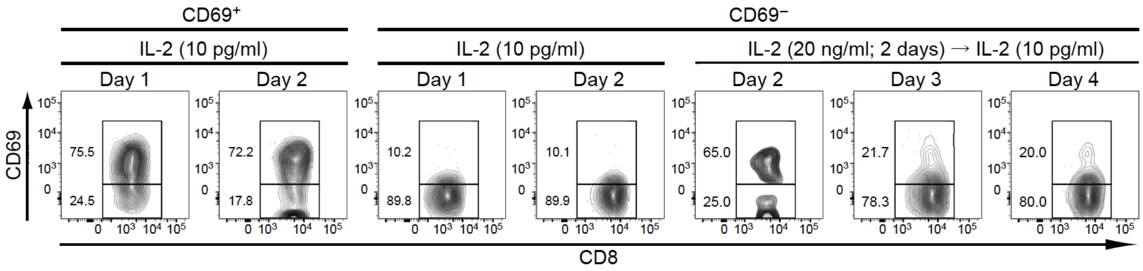

Supplementary Figure 2. CD69 expression on TILs is retained during culture. Mononuclear cells were isolated by density gradient centrifugation with Ficoll-Paque PLUS (GE Healthcare) from single-cell suspensions prepared from tumour tissue and stained for CD69. $CD69^+$ and $CD69^-$ lymphocytes were sorted using a FACSAria and cultured with low-dose IL-2 (10 pg/ml) for 2 days. Isolated $CD69^-$ cells were also stimulated with high-dose IL-2 (20 ng/ml) for 2 days and then cultured with low-dose IL-2 (10 pg/ml) for 2 days. CD69 expression on $CD8^+$ T cells was determined at indicated days by flow cytometry. Numbers adjacent to the outlined areas indicate the percentage of cells in each.

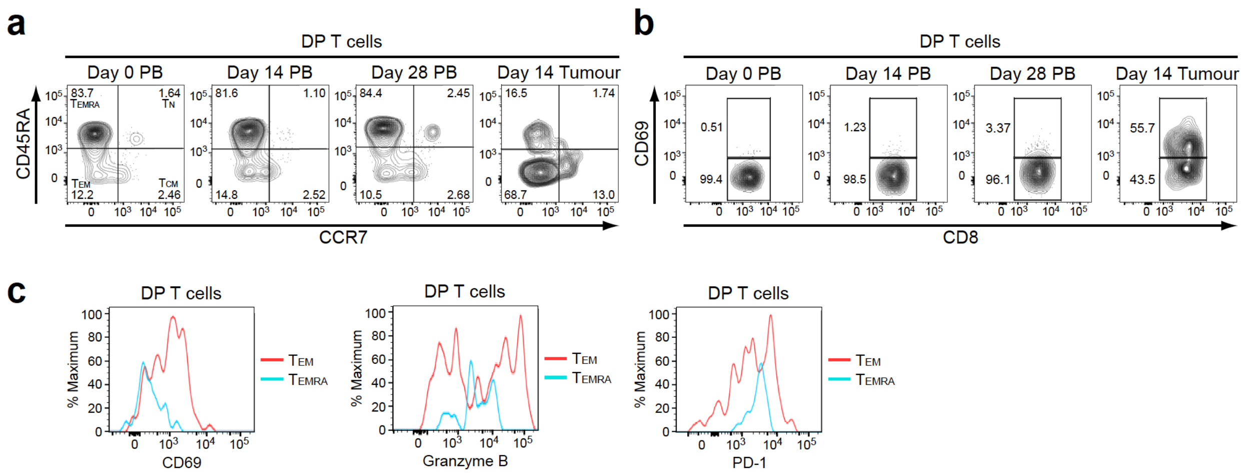

Supplementary Figure 3. DP T cell phenotype in cynomolgus macaques. (**a**) Expression of CCR7 and CD45RA in DP T cells from PB and tumour tissue. Blood was obtained at days 0, 14 and 28, and the tumour was resected at day 14 after tumour transplantation. The T_N, T_{CM}, T_{EM} and T_{EMRA} subsets were determined as CD45RA$^+$CCR7$^+$, CD45RA$^-$CCR7$^+$, CD45RA$^-$CCR7$^-$ and CD45RA$^+$CCR7$^-$, respectively. Numbers in quadrants indicate the percentage of cells in each. (**b**) Expression of CD69 in DP T cells from PB and tumour tissue. Numbers adjacent to the outlined areas indicate the percentage of cells in each. (**c**) Expression of CD69, granzyme B and PD-1 in DP T_{EM} and T_{EMRA} cells from tumours.
